# Constant output characteristics and design methodology of double side LC compensated capacitive power transfer

**DOI:** 10.1038/s41598-022-06577-x

**Published:** 2022-02-17

**Authors:** Qiao Xiong, Ying Shao, Pan Sun, Jun Sun, Enguo Rong, Yan Liang

**Affiliations:** College of Electrical Engineering, Naval University of Engineering, Wuhan, 430000 Hubei China

**Keywords:** Electrical and electronic engineering, Energy infrastructure

## Abstract

Capacitive power transfer (CPT) has been verified to be capable of transferring a power level as high as inductive power transfer (IPT) recently, and has its own merits. It is a well complement of IPT in near-field wireless power transfer (WPT). This paper gives a newly designed method of realizing both constant output voltage (COV) and constant output current (COC) modes of double side LC compensated CPT. Firstly, through analysis of basic circuit characteristics, the conditions for both of the two modes are deduced theoretically. Especially, one merit of the method is that the conditions indicate a very clear relationship between the compensation components forming resonant tanks. Another merit is that the couple capacitors also participate in resonant tanks. Different from the COV mode, the COC mode can theoretically reach zero phase angle condition simultaneously. Based on these conditions, the parameter design methodology is proposed. Besides, an efficient model of double side LC compensated CPT is built, and the optimum load is calculated theoretically to guide the design course. Finally, the results of both simulations and experiments demonstrate high consistency with the theoretical analysis.

## Introduction

In the past few years, inductive power transfer (IPT) utilizing inductive coils is most widely studied as a conventional wireless power transfer (WPT) technology, though some technical obstacles are difficult to overcome, like bulky couple structure, eddy current loss in nearby conductive objectives, weak performance when misaligned^1,2^. However, capacitive power transfer (CPT**)** has been verified to be effective and capable of transferring a power level as high as IPT in a significant transfer distance^3^. In 2015, it is reported that CPT can transfer very high power density at 1.1 W/mm^2^ at RF 100 MHz frequency^4^, and another research reported a prototype that can transfer power in kW level at 15 cm transfer distance and high efficiency over 90%^5^, Since then, increasing attention has been attracted and explosive achievements have been made in CPT research field^6^. Compared with the earlier and more widely studied IPT, CPT has its own merits like flexible and low cost in coupler, good performance when misalignment exists, low eddy current loss induced in nearby metal objects and no requirement for EMI shielding^6–8,13,14,19,27^. Thus, CPT has the potential of applying in some special occasions like in plantable medical appliance^9,10^, rotating machine^11^ or seawater^12,13^. Another breakthrough in CPT technology is that a simplified equivalent model for four-plate coupler was built^14^, based on which further studies of compensation net can be done, to exploit the basic characteristics of a CPT system.

In a CPT system, the significance of compensation net lies in that it can directly define the system transfer property and performance, such as boost the voltage between couple plates to overcome the transfer distance, compensate the system reaction power to make a high power factor, set the voltage or current gain of the system, and consume low energy to ensure a high transfer efficiency. There are plenty of researches focusing on compensation net^5–8,15–28,30–33^. The most commonly used compensation nets in CPT range from two-order to four-order. High-order compensation net is often needed to enhance the transfer capability limited by the small value of couple capacitance, like four order compensation net has been adopted^15,16^. However, some defects will be brought about by high-order compensation net, such as increasing the system complexity, high voltage stress on the compensation components, etc. As a result, we would like to further study the transfer property of the 2-order double side LC compensated CPT in this work.

As we know, constant transfer property is one of the basic requirement for a WPT system. A method by changing the operation frequency to achieve constant power and efficiency when the couple situation varies is proposed^17^, but the close-loop control will increase the complexity and cost of CPT system. In some researches, CPT with double side LC compensation net is analyzed^18,19,21,25,30^, especially both constant output voltage (COV) and constant output current (COC) conditions are proposed based on the deduction of voltage or current gain^19^. However, there are defects in the existing research references. For instance, conditions for both the COV mode and COC mode attribute to operation frequencies different from the intrinsic resonant frequency of the LC compensation net in either the primary side or secondary side, leading to a vague relationship between the resonant components. Furthermore, the couple efficiency in an ordinary CPT system is generally very low, leading to a very vicinal operation frequencies for COV and COC modes, which would be also in high accordance with the intrinsic resonant frequency of the LC compensation net. However, the operation frequency can be hardly defined precisely because it is always affected by the manufacture deviation or test deviation of the compensation components and the parasitic reactance of circuit. These will finally induce a trouble in defining the operating frequency in practice. Therefore, this paper aims at disclosing a more intuitive and practical method of decoupling the load resistance, to address the aforementioned problems.

Four categories for constant output are summarized^20^, although they address IPTs, they can also be adopted in CPTs. Accordingly, this paper proposes the COV and COC mode conditions by analyzing the basic circuit characteristics. Then, an efficient model is established and an optimum load resistance is theoretically deduced. Based on these, the design methodology of system parameters is suggested. Both simulation and experiment are carried out to verify them. Finally, three practical issues are discussed. It is demonstrated that the method proposed in this paper is more precise and practical.

## Results

### Theoretical model and analysis

**Figure 1:**
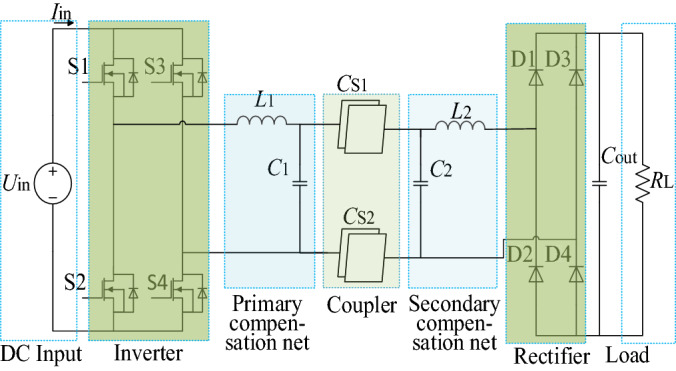
Schematic of a double side LC compensated CPT. (Created by ‘Microsoft Office Visio 2013’ url: https://www.microsoft.com/zh-cn/microsoft-365/previous-versions/microsoft-visio-2013).

A double side LC compensated CPT mainly falls into seven parts, as shown in Fig. 1. Supposing that the input power is a DC source, it should be changed into a high frequency AC that can resonant in the tank of the primary LC compensation net, triggering a high flux electric field between the couple plates, and causing a displacement current from the emission plates of the coupler to the receiving plates. Then, the electricity power going through the coupler will be stored temporarily in the resonant tank of the secondary LC compensation net, providing a source to feed the rectifier and drive the load.

**Figure 2:**
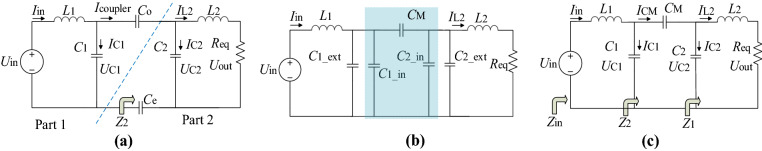
Equivalent model of CPT with double side LC compensation net. (**a**) Equivalent circuit with no cross coupling. (**b**) Simplified circuit model with equivalent coupler model. (**c**) Further simplified circuit model of (**b**). (Created by ‘Microsoft Office Visio 2013’ url: https://www.microsoft.com/zh-cn/microsoft-365/previous-versions/microsoft-visio-2013).

The proposed CPT can be simplified as Fig. 2a. The output of the inverter is treated as a high frequency input voltage in Fig. 2a. When the horizontal distance between plates is significant enough, the cross-couple capacitance can almost be negligible and the couple capacitance is just the capacitance formed by each pair of plates. *C*_o_ is defined as the capacitance formed by odd plates P1 and P3, and *C*_e_ is the capacitance formed by plates P2 and P4. When taking consideration of the cross-couple capacitance between each two plates, the equivalent model of the coupler is proposed in reference^14^, shown in the blue pane in Fig. 2b. The load resistance sourced by the rectifier with a parallel capacitor filter can be equivalent to *R*_eq_ by 8*R*_L_/π^2^ ^19^. In Fig. 2b, suppose *C*_1_ = *C*_1_ext_ + *C*_1_in_ and *C*_2_ = *C*_2_ext_ + *C*_2_in_. Thus, a further simplified equivalent circuit in Fig. 2c is derived.

### Analysis of constant output characteristics

Almost all appliances use electricity as power supply expect a constant input to gain a normal rated power. In this part, the constant output characteristics, including COC and COV, will be exploited based on the equivalent models given in the former part.

**Figure 3:**
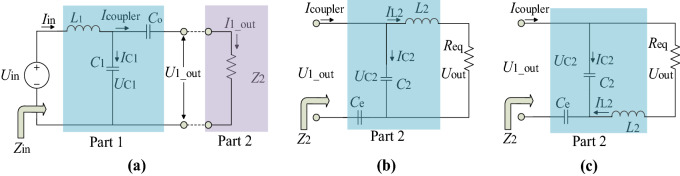
Symmetric of double side LC compensated CPT and its division of two parts. (**a**) Part 1 of the circuit, with equivalent resistance of Part 2. (**b**) Part 2 with output voltage of Part 1 as its input. (**c**) A change-form of (**b**). (Created by ‘Microsoft Office Visio 2013’ url: https://www.microsoft.com/zh-cn/microsoft-365/previous-versions/microsoft-visio-2013).

A. *COV mode* The schematic in Fig. 2a can be divided in two symmetrical parts by the dashed blue line shown in Fig. 3a,b. Figure 3c is a change form of Fig. 3b, with only a position change in *L*_2_. Supposing that the operation frequency of the system is ω, the voltage *U*_1_out_ in Fig. 3a can be defined by (1), and the voltage *U*_out_ in Fig. 3b can be defined by (2).
  1 $$U_{{{\text{1\_out}}}} = \frac{{j\omega C_{{\text{O}}} Z_{2} }}{{\Phi_{1} + j\omega C_{{\text{O}}} Z_{2} (1 - \omega^{2} L_{1} C_{1} )}}U_{{{\text{in}}}} ,$$
  2 $$U_{{{\text{out}}}} = \frac{{j\omega C_{{\text{e}}} R_{{{\text{eq}}}} }}{{\Phi_{2} + j\omega R_{{{\text{eq}}}} (C_{2} + C_{{\text{e}}} )}}U_{{{\text{1\_out}}}} .$$

In (1) and (2), $$\Phi_{1} = 1 - \omega^{2} L_{1} (C_{1} + C_{{\text{O}}} ),\Phi_{2} = 1 - \omega^{2} L_{2} (C_{2} + C_{{\text{e}}} ).$$

Combine (1) and (2), a special voltage gain can be derived by (4) when (3) is met. It is clear that the voltage gain *G*_V_ of the CPT has no relationship with *R*_eq_ in (4), indicating a constant voltage output of CPT. Thus, Eq. (3) is the condition for COV mode, and it indicates that *L*_1_ is in resonance with the parallel capacitance of *C*_1_ and *C*_o_, and *L*_2_ is in resonance with the parallel capacitance of *C*_2_ and *C*_e_. The resonant tanks are marked in blue panes in Fig. 3. According to (4), the output power is defined by (5).

3 $$\left\{ {\begin{array}{*{20}c} {\Phi_{1} = 1 - \omega^{2} L_{1} (C_{1} + C_{{\text{O}}} ) = 0} \\ {\Phi_{2} = 1 - \omega^{2} L_{2} (C_{2} + C_{{\text{e}}} ) = 0,} \\ \end{array} } \right.$$

4 $$G_{{\text{V}}} = \frac{{U_{{{\text{out}}}} }}{{U_{{{\text{in}}}} }} = \frac{{C_{{\text{e}}} (C_{{1}} + C_{{\text{o}}} )}}{{C_{{\text{o}}} (C_{{2}} + C_{{\text{e}}} )}} = \frac{{\lambda_{1} + 1}}{{\lambda_{2} + 1}}.$$

**Figure 4:**
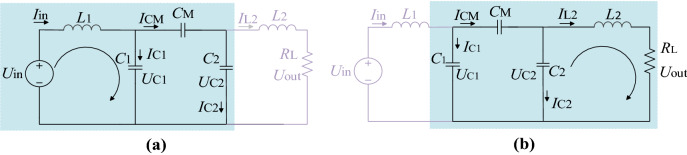
Resonant tanks in CPT. (**a**) Resonant tank in primary side. (**b**) Resonant tank in secondary side. (Created by ‘Microsoft Office Visio 2013’ url: https://www.microsoft.com/zh-cn/microsoft-365/previous-versions/microsoft-visio-2013).

In (4), *C*_1_ = λ_1_*C*_o_ and *C*_2_ = λ_2_*C*_e_.

5 $$P_{{{\text{R}}_{{\text{L}}} }} = \frac{{U_{{{\text{out}}}}^{2} }}{{R_{{\text{L}}} }} = \frac{{C_{{\text{e}}}^{{2}} (C_{1} + C_{{\text{o}}} )^{2} U_{{{\text{in}}}}^{{2}} }}{{C_{{\text{o}}}^{{2}} (C_{2} + C_{{\text{e}}} )^{2} R_{{\text{L}}} }} = \left( {\frac{{\lambda_{1} + 1}}{{\lambda_{2} + 1}}} \right)^{2} \frac{{U_{{{\text{in}}}}^{{2}} }}{{R_{{\text{L}}} }}.$$

B. In Fig. 2c, resistance Z_1_, Z_2_, Z_3_ can be expressed by (6), (7) and (8). Combining (6), (7) and (8), *Z*_2_ and *Z*_in_ can be derived by (9) and (10). To achieve zero phase angle, the input impedance should be purely resistant. In this case, the vector of numerator and denominator in Eq. (10) should have the same angle, a special situation is that the condition in (11) is met. Equation (11) can be changed into (12). From (12), it is easy to find that the inductance *L*_1_ and a combination of capacitance that *C*_2_ in serial with *C*_M_, then in parallel with *C*_1_, form a resonant tank in the primary part. Similarly, *L*_2_ and *C*_1_, *C*_2_, *C*_M_ form another resonant tank in the secondary part. The resonant tanks are exhibited in Fig. 4. The equivalent capacitance of the coupler takes part in both primary and secondary resonant tanks. Substituting (11) into (10), we can get (13).

6 $$Z_{1} = (j\omega L_{2} + R_{{{\text{eq}}}} )\parallel \frac{1}{{j\omega C_{2} }} = \frac{{j\omega L_{2} + R_{{{\text{eq}}}} }}{{1 - \omega^{2} L_{2} C_{2} + j\omega R_{{{\text{eq}}}} C_{2} }},$$

7 $$Z_{2} = \left( {Z_{1} + \frac{1}{{j\omega C_{M} }}} \right)\left\| {\frac{1}{{j\omega C_{1} }}} \right.,$$

8 $$Z_{{{\text{in}}}} = j\omega L_{1} + Z_{2} ,$$

9 $$Z_{2} = \frac{{1 - \omega^{2} L_{2} (C_{2} + C_{{\text{M}}} ) + j\omega (C_{2} + C_{{\text{M}}} )R_{{{\text{eq}}}} }}{{j\omega \Delta_{2} - \omega^{2} (C_{1} C_{2} + C_{1} C_{{\text{M}}} + C_{2} C_{{\text{M}}} )R_{{{\text{eq}}}} }},$$

10 $$Z_{{{\text{in}}}} = \frac{{1 - \omega^{2} L_{2} (C_{2} + C_{M} ) - \omega^{2} L_{1} \Delta_{2} + j\omega R_{{{\text{eq}}}} \Delta_{1} }}{{ - \omega^{2} R_{{{\text{eq}}}} (C_{1} C_{2} + C_{1} C_{M} + C_{2} C_{M} ) + j\omega \Delta_{2} }}.$$

In (9) and (10), $$\left\{ \begin{gathered} \Delta_{1} = (C_{2} + C_{M} ) - \omega^{2} L_{1} (C_{1} C_{2} + C_{1} C_{M} + C_{2} C_{M} ), \hfill \\ \Delta_{2} = (C_{1} + C_{M} ) - \omega^{2} L_{2} (C_{1} C_{2} + C_{1} C_{M} + C_{2} C_{M} ). \hfill \\ \end{gathered} \right.$$

11 $$\left\{ {\begin{array}{*{20}c} {\Delta_{1} = 0} \\ {\Delta_{2} = 0,} \\ \end{array} } \right.$$

12 $$\left\{ \begin{gathered} \omega L_{1} = \frac{1}{{\omega \left( {C_{1} + \frac{{C_{2} C_{M} }}{{C_{2} + C_{M} }}} \right)}} \hfill \\ \omega L_{2} = \frac{1}{{\omega \left( {C_{2} + \frac{{C_{1} C_{M} }}{{C_{1} + C_{M} }}} \right)}}, \hfill \\ \end{gathered} \right.$$

13 $$Z_{{{\text{in}}}} = \frac{{[\omega^{2} L_{1} (C_{1} + C_{{\text{M}}} ) - 1]L_{2} }}{{(C_{1} + C_{{\text{M}}} )R_{{{\text{eq}}}} }}.$$

Then, voltage of *C*_1_, *C*_2_, and *U*_out_ can be expressed by (14), (15) and (16), respectively. Combining (6), (13), (14), (15) and (16), the output voltage can be derived from (17). Current of load resistance *R*_L_ can be expressed by (18) when *C*_1_ = *k*_1_*C*_M_ and *C*_2_ = *k*_2_*C*_M_. It can be intuitively seen that the output current of the proposed CPT has no relationship with the load resistance. The power on the load resistance can be defined by (19), which demonstrates that the output power is defined positively by the square of the angular frequency ω and the load resistance.

14 $$U_{{{\text{C}}_{{1}} }} = U_{{{\text{in}}}} - j\omega L_{1} \frac{{U_{{{\text{in}}}} }}{{Z_{{{\text{in}}}} }},$$

15 $$U_{{{\text{C}}_{{2}} }} = \frac{{Z_{{1}} }}{{Z_{{1}} + \frac{1}{{j\omega C_{{\text{M}}} }}}}U_{{{\text{C}}_{{1}} }} ,$$

16 $$U_{{{\text{Out}}}} = \frac{{R_{{{\text{eq}}}} }}{{R_{{{\text{eq}}}} + j\omega L_{{2}} }}U_{{{\text{C}}_{{2}} }} ,$$

17 $$U_{{{\text{out}}}} = \frac{{j\omega C_{{\text{M}}} R_{{{\text{eq}}}} }}{{1 - \omega^{2} L_{2} (C_{2} + C_{{\text{M}}} )}}U_{{{\text{in}}}} ,$$

18 $$I_{{{\text{R}}_{{\text{L}}} }} = \frac{{U_{{{\text{out}}}} }}{{R_{{\text{L}}} }} = - j\frac{8}{{\pi^{2} }}\omega \left( {\frac{{C_{{1}} C_{2} }}{{C_{{\text{M}}} }} + C_{{1}} + C_{2} } \right)U_{{{\text{in}}}} = - j\frac{8}{{\pi^{2} }}\omega (k_{{1}} k_{2} + k_{{1}} + k_{2} )C_{{\text{M}}} U_{{{\text{in}}}} ,$$

19 $$P_{{{\text{R}}_{{\text{L}}} }} = \left( {\frac{8}{{\pi^{2} }}} \right)^{2} \omega^{2} (k_{{1}} k_{2} + k_{{1}} + k_{2} )^{2} C_{{_{{\text{M}}} }}^{2} U_{{{\text{in}}}}^{{2}} R_{{\text{L}}} .$$

C. *Analysis of DC–DC efficiency* From the conclusion above, it is known that the zero phase angle can be achieved in COC mode, making zero voltage switching (ZVS) possible to realize. However, ZVS cannot be achieved in COV mode. Therefore, the COC mode is chosen to analyze the system efficiency. The method for COV mode is similar.

**Figure 5:**
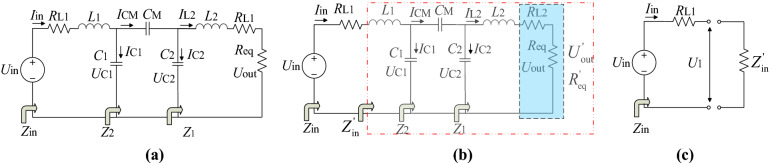
Efficiency model of COC mode. (**a**) Circuit with *R*_L1_ and *R*_L2_. (**b**) Replace R_L2_ and *R*_eq_ with *R*′_eq_. (**c**) Replace circuit in the red dash line frame in (**b**) with *Z*′_in_. (Created by ‘Microsoft Office Visio 2013’ url: https://www.microsoft.com/zh-cn/microsoft-365/previous-versions/microsoft-visio-2013).

The parasitic resistance of each component in the system is needed to be analyzed when establishing the efficiency model. However, the impact of parasitic resistance from couple capacitors can be limited by choosing high quality factor capacitors *C*_1_, *C*_2_. The non-core inductance winded by litz wire should be adopted to reduce iron-core loss and serial parasitic resistance caused by skin effect when the system works at a high frequency. But a long litz wire will bring non-negligible parasitic resistance. Parasitic resistance also exists in the inverter and rectifier, known as turn-on resistance. In addition, energy loss in the inverter and rectifier also includes switching loss that is much more complex than the turn-on loss, and it has already been well studied^34^. Here, ZVS is supposed to be realized and the switching loss is negligible. Supposing that the parasitic resistance of inverter and *L*_1_ contributes to *R*_L1,_ rectifier and *L*_2_ contributes to *R*_L2_, shown in Fig. 5a. To simplify the analysis, *R*_L2_ and *R*_eq_ can be considered as a whole, *R*′_eq_. In Fig. 5b, circuit in the red dash line frame can be treated as an impedance *Z*′_in_ in Fig. 5c. Based on the assumption above, the system power loss can be calculated by (20). Therefore, the system efficiency can be calculated by (21). The derivation of *η* can be expressed in (22). It can be easily defined that the system efficiency has a maximum optimum value *η*_max_ when *R*′_eq_ has the value *R*′_eq_opt_, calculated by (23).

20 $$\left\{ \begin{array}{*{20}l} P_{{R_{{L1}} }} = I_{{{\text{in}}}}^{2} R_{{L1}} = \left( {\frac{{\omega ^{2} \Delta ^{2} R_{{{\text{eq}}}}^{'} U_{{{\text{in}}}} }}{{C_{{\text{M}}}^{2} + \omega ^{2} R_{{L1}} \Delta ^{2} R_{{{\text{eq}}}}^{'} }}} \right)^{2} R_{{L_{1} }} \\ P_{{R_{{L2}} }} = I_{{{\text{out}}}}^{2} R_{{L2}} = \left( {\frac{{8\omega \Delta }}{{\pi ^{2} C_{{\text{M}}} }}\frac{{C_{{\text{M}}}^{2} }}{{C_{{\text{M}}}^{2} + \omega ^{2} R_{{L1}} \Delta ^{2} R_{{{\text{eq}}}}^{'} }}U_{{{\text{in}}}} } \right)^{2} R_{{L2}} = \frac{{64\omega ^{2} \Delta ^{2} C_{{\text{M}}}^{2} U_{{{\text{in}}}}^{2} R_{{L2}} }}{{\pi ^{4} (C_{{\text{M}}}^{2} + \omega ^{2} R_{{L1}} \Delta ^{2} R_{{{\text{eq}}}}^{'} )^{2} }}, \\ \end{array} \right.$$

**Figure 6:**
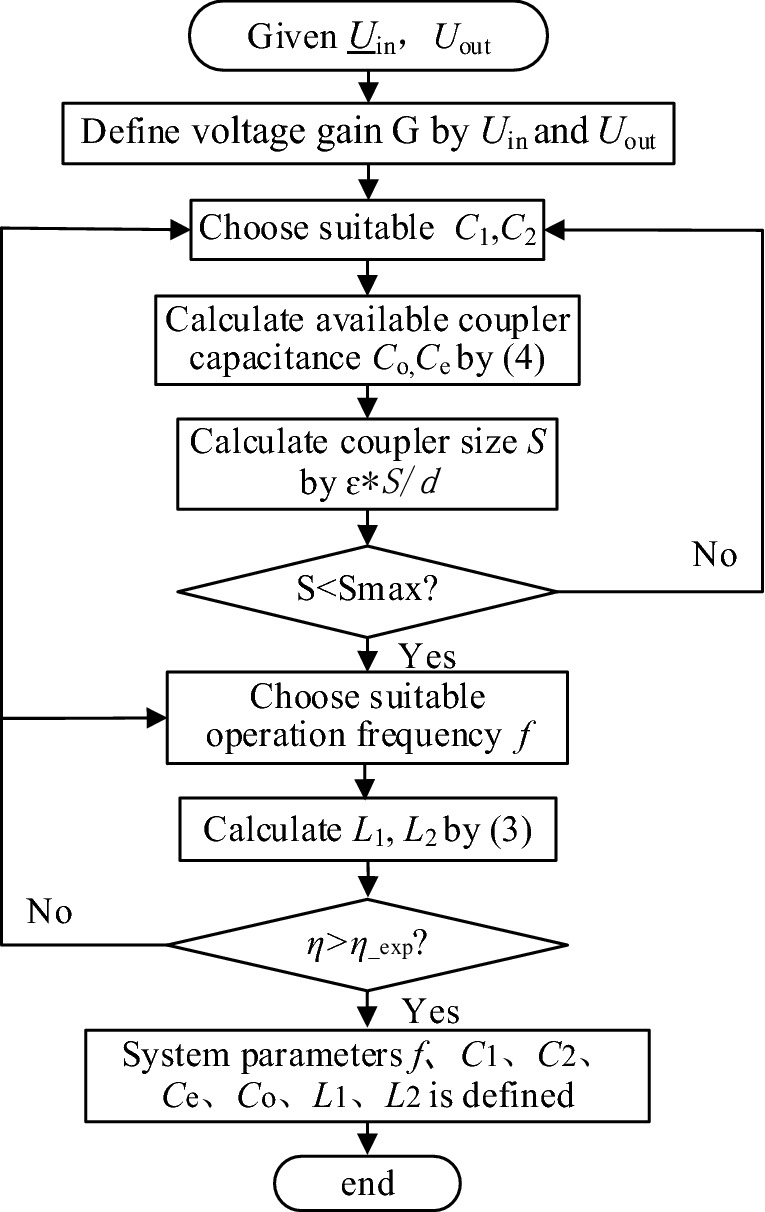
Diagram of parameter design procedure for a COV system. (Created by ‘Microsoft Office Visio 2013’ url: https://www.microsoft.com/zh-cn/microsoft-365/previous-versions/microsoft-visio-2013).

In (20), Δ = *C*_1_*C*_2_ + *C*_1_*C*_M_ + *C*_2_*C*_M_.

21 $$\eta = 1 - \frac{{P_{{{\text{loss}}}} }}{{P_{{{\text{in}}}} }} = 1 - \frac{{P_{{{\text{R}}_{{{\text{L1}}}} }} + P_{{{\text{R}}_{{{\text{L2}}}} }} }}{{P_{{{\text{in}}}} }} = \frac{{CR_{{{\text{eq}}}}^{\prime } - B}}{{AR_{{{\text{eq}}}}^{{\prime 2}} + CR_{{{\text{eq}}}}^{\prime } }}.$$

In (21), $$\left\{ \begin{gathered} A = \pi^{4} \omega^{2} \Delta^{2} R_{{L_{1} }} , \hfill \\ B = 64C_{{\text{M}}}^{2} R_{L2} , \hfill \\ C = \pi^{4} C_{{\text{M}}}^{2} . \hfill \\ \end{gathered} \right.$$

22 $$\frac{{\partial \eta }}{{\partial R_{{{\text{eq}}}} }} = - \frac{{ACR_{{{\text{eq}}}}^{{\prime 2}} - 2ABR_{{{\text{eq}}}}^{\prime } - BC}}{{(AR_{{{\text{eq}}}}^{{\prime 2}} + CR_{{{\text{eq}}}}^{\prime } )^{2} }},$$

23 $$\left\{ \begin{gathered} R_{{{\text{eq\_opt}}}}{^{\prime}} = \frac{{AB + \sqrt {A^{2} B^{2} + ABC^{2} } }}{AC} \hfill \\ \eta_{\max } = \frac{{C^{2} }}{{2AB + C^{2} + 2\sqrt {A^{2} B^{2} + ABC^{2} } }}. \hfill \\ \end{gathered} \right.$$

### Design methodology

According to the analysis above, the general design methodology is discussed in this part. Before designing a CPT, the basic demands like nominal input, rated output and physical dimensions allowed for the coupler should be acquired.

A. *Parameter design for COV mode* If the rated voltage demand U_in_, U_out_ is given, the voltage gain is clear. Then, suitable compensation capacitors *C*_1_ and *C*_2_ can be chosen. So the coupler capacitance *C*_o_, *C*_e_ can be defined by (4). Then, the coupler size can be calculated by ε*S/d. The sizes of the couple plates are often restricted by the available volume of a certain appliance. Thus, to gain a considerable capacitance, a trade-off between the transfer distance and the size of the coupler should be made. If the calculated coupler size is bigger than the allowed range, we should return to choose *C*_1_ and *C*_2_. Otherwise, the procedure will continue to choose a suitable operation frequency *f*. Then the value of inductors *L*_1_ and *L*_2_ can be calculated. Volume and parasitic resistance of *L*_1_, *L*_2_ is another constraint. Because the parasitic equivalent serial resistance *R*_L1_ and *R*_L2_ is positively related to the value of *L*_1_, *L*_2_. If the efficiency *η* is not higher than the expected or designed value *η*_exp_, the procedure will go back to choose *C*_1_ and *C*_2_, or *f*. After defining *L*_1_, *L*_2_, MOSFETs for the inverter and diodes for the rectifier should be chosen according to the frequency, voltage, and current. The procedure of parameter design for a COV CPT system is concluded in Fig. 6.
B. *Parameter design for COC mode* The procedure of parameter design for COC CPT system is similar to that of a COV system. However, the difference is that the output current is directly proportional to the angular frequency ω, so when choosing *C*_1_, *C*_2_ and *f*, a trade-off should be made between them. Another trade-off is between the coupler size or transfer distance and the couple capacitance. Especially the output current and power is negatively related to the mutual couple capacitance *C*_M_. However, according to the (21), the system efficiency is in positive relationship with *C*_M_. A smaller couple capacitance will also require a higher operation frequency or larger resonant inductor, which will trigger a high voltage stress between plates.

These design methodology will be verified in the next section by simulation and experiments. The DC-to-DC efficiency of the system will also be verified.

### Simulations and experiments

The constant output characteristics and design methodology are verified through MATLAB simulations and experiment tests. Table 1 gives the parameters designed by the proposed methodology.

**Table 1:** Main parameters designed for testing.

Mode	Demands		Compensation components		Coupler size	
COV	*V*_in_	60 v	*L*_1_ (*L*_2_)	33.8uH	*C*_o_ (*C*_e_)	2850 pF
	*G*_v_	1	*C*_1_ (*C*_2_)	5.5nF	*W *(*l*)	265 mm
COC	*V*_in_	60 v	*L*_1_ (*L*_2_)	42.8uH	*d*	0.05 mm
	*G*_i_	0.05	*C*_1_ (*C*_2_)	5.5nF	*w*_0_	600 mm
Operation freq *f*			300 kHz		*R*_L_	5 ~ 55 Ω

**Figure 7:**
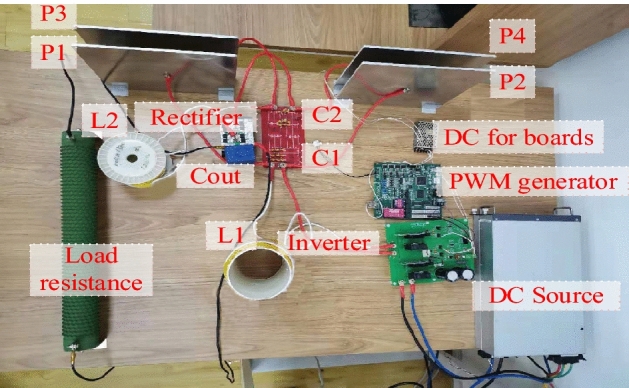
Experiment platform. Taken by the first author Qiao Xiong,through the digital camera of mobilephone, and the descriptive text is added by the software “Microsoft Office Visio 2013” url: https://www.microsoft.com/zh-cn/microsoft-365/previous-versions/microsoft-visio-2013.

Firstly, The system parameters in Table 1 are used to calculate the output characteristics of the system directly in MATLAB. Then, an experiment prototype in Fig. 7 is built for further verification. In this prototype, a DSP board serves as PWM generator, N-Channel SiC MOSFET LSIC1MO120E0080 is adopted to form the full-bridge inverter, litz wire is used for all connections in the high frequency part to minimize the loss caused by skin effect. The coupler is made by four aluminum plates with the size shown in the Table 1. Gap distance between each pair of plates is filled with a plastic paper (0.05 mm in thickness) to enhance the capacitance of the coupler. In fact, this experiment prototype aims at verifying the constant output characteristics of the suggested system, so the affection from transfer distance is not studied. Numerical quantity of each component in the experiment platform is measured by high-precision LCR Meter, shown in Table 2.

**Table 2:** Numerical quantity tested by LCR meter at 300 kHz.

Component name		Value (pF or uH)	Quality factor	R − X (Λ)
*C*_o_		2849.85	954.7	0.195 − 186.16
*C*_e_		2858.74	672.7	0.276 − 185.67
*C*_1_		5478.65	1126.0	0.08600 − 96.835
*C*_2_		5493.73	1095.9	0.08812 − 96.570
*L*_1_	COV	33.971	587.5	0.109 + 64.034
	COC	42.905	680.6	0.118 + 80.306
*L*_2_	COV	33.829	861.7	0.074 + 63.766
	COC	42.8085	1871.2	0.042 + 78.592

**Table 3:** Different groups of compensation parameters.

Component name		Value (pF or uH)	Parasitic resistance (Λ)	Corresponding curve in Fig. 17
*C*_o_		2849.85	0.195	–
*C*_e_		2858.74	0.276	–
Group1	*C*_1_	5478.65	0.086	Data 1
	*C*_2_	5493.73	0.088	
	*L*_1_	42.905	0.118	
	*L*_2_	42.8085	0.042	
	*G*_1_	0.0499	–	
Group2	*C*_1_	2980.3	0.116	Data 2
	*C*_2_	3030.6	0.099	
	*L*_1_	71.529	0.615	
	*L*_2_	70.721	0.581	
	*G*_1_	0.0191	–	
Group3	*C*_1_	4868.53	0.85	Data 3
	*C*_2_	4685.41	0.824	
	*L*_1_	47.393	0.32	
	*L*_2_	48.812	0.245	
	*G*_1_	0.0397	–	
Group4	*C*_1_	1727.14	0.065	Data 4
	*C*_2_	1714.61	0.069	
	*L*_1_	113.34	1.288	
	*L*_2_	113.82	1.345	
	*G*_1_	0.0085	–

**Figure 8:**
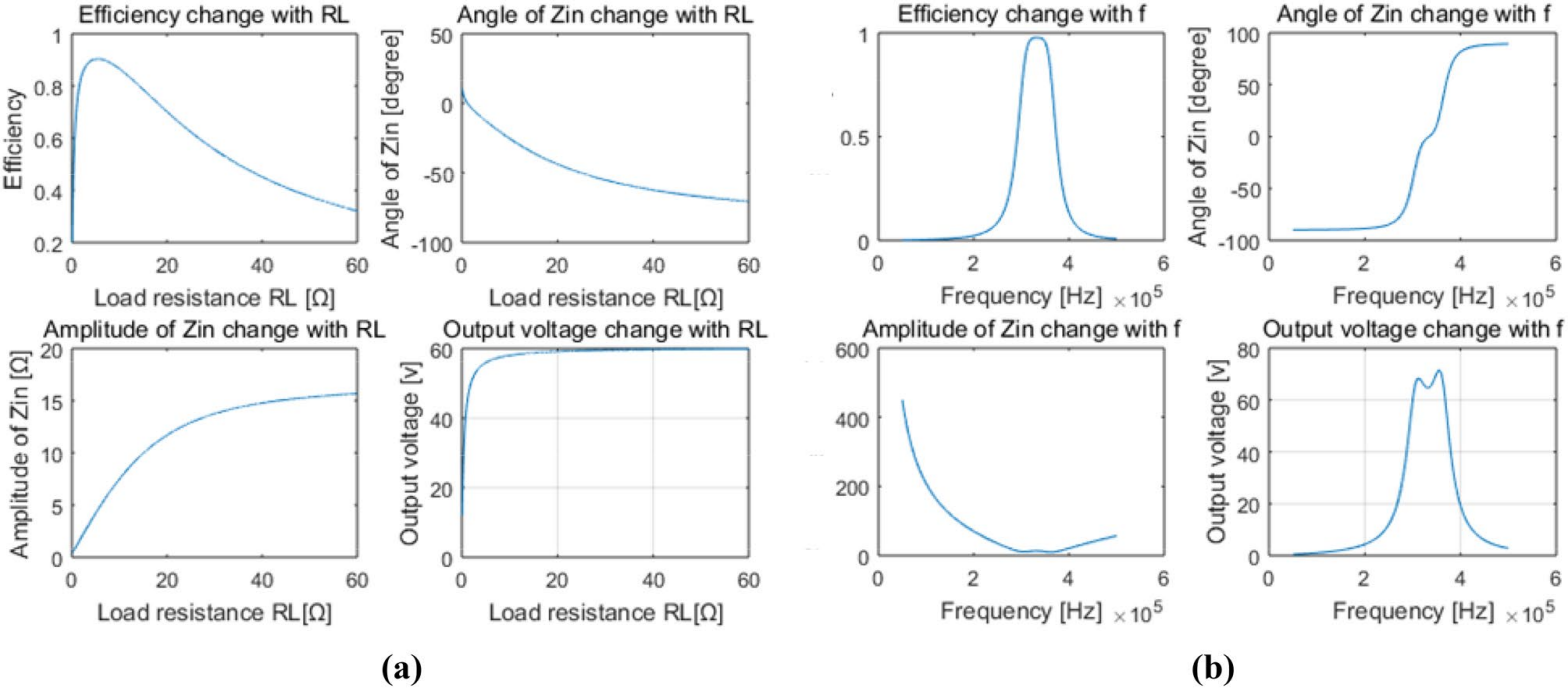
Main characteristics change with RL (**a**) and frequency (**b**) of the proposed system working in constant output voltage mode, derived from MATLAB. (Created by “matlab R2016a” url: https://ww2.mathworks.cn/products/matlab.html).

A. *COV mode* MATLAB simulation results are presented in Fig. 8. It is demonstrated that the system efficiency increases firstly and then decreases with the increase of *R*_L_ in Fig. 8a, and the optimum load resistance is about 5Ω. The maximum efficiency is 90.4%, which is in good accordance with the theoretical analysis. Both the amplitude and absolute value of angle of the total input impedance *Z*_in_ increase with *R*_L_, but the trends become slow. Especially, the angle of *Z*_in_ is negative, which indicates *Z*_in_ is capacitive, and zero phase angle cannot be achieved. However, the output voltage shows a constant value of about 60 V when *R*_L_ is above 5Ω. Figure 8b is the response of the system against the system frequency when *R*_L_ is 20Ω. Curves in Fig. 8b indicate that the compensation net is intrinsically a band-pass filter.

**Figure 9:**
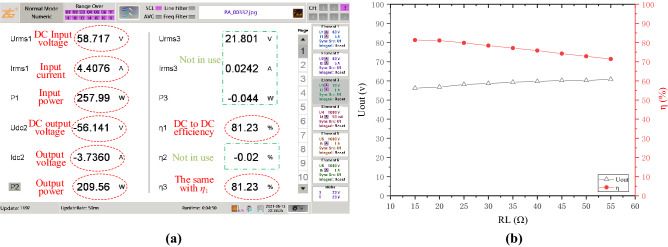
Experiment result in COV mode. (**a**) DC input and DC output power tested by ZLG Power Analyzer. (**b**) The tested output voltage and efficiency change with *R*_L_. [(**a**) is obtained by power analyzer, and the descriptive text is added by the “Microsoft Office Visio 2013” url: https://www.microsoft.com/zh-cn/microsoft-365/previous-versions/microsoft-visio-2013, (**b**) is created by “origin 2018”, url: https://www.0daydown.com/tag/originpro-2018].

Experiment result in Fig. 9a is taken by ZLG Power Analyzer. Due to the voltage drop caused by the parasitic resistance of switch devices, the tested output voltage is slightly lower than simulation. It also shows an DC to DC efficiency of 81.23% when *R*_L_ is 15Ω, close to Fig. 8a. The system efficiency is not so high, due to the significant angle of the total input impedance, and ZVS condition can’t be achieved, either. The black curve in Fig. 9b shows the output voltage changes with *R*_L_, which indicates a tiny increase when the load resistance increasing, due to the decrease of current level in the whole system. The red curve in Fig. 9b exhibits that the DC-to-DC efficiency of the COV system decreases with the increase of *R*_L_. This phenomenon is in good accordance with the trend of the total input impedance angle shown in Fig. 8a.

**Figure 10:**
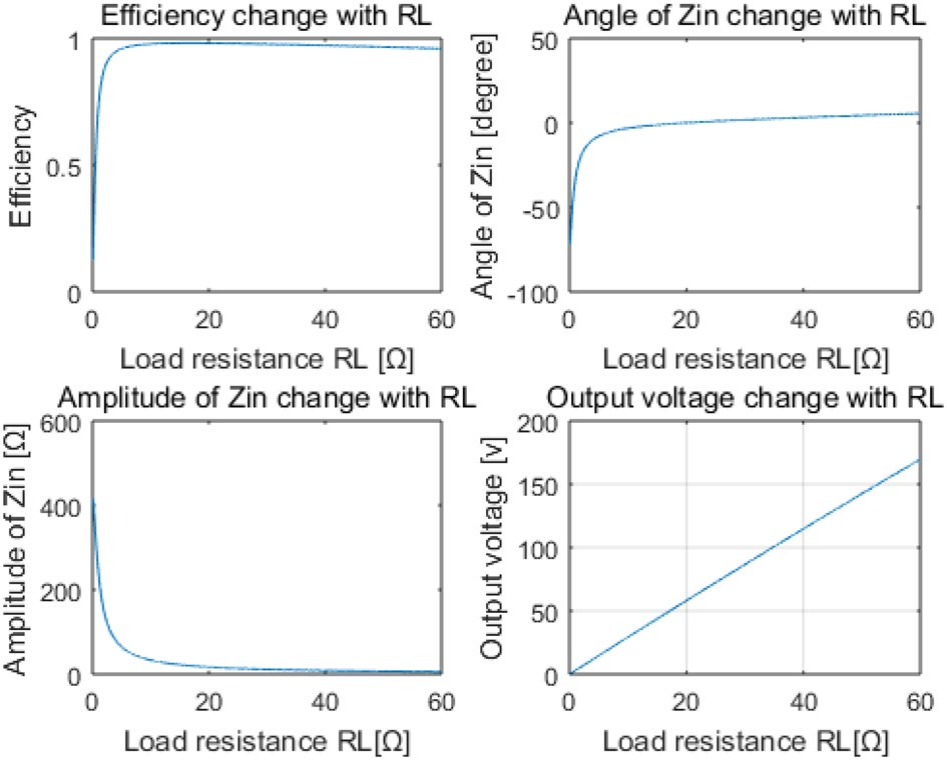
Main output characteristics of the proposed system working in COC mode, derived from MATLAB. (Created by “matlab R2016a” url: https://ww2.mathworks.cn/products/matlab.html).

B. *COC mode* Curves drawn by MATLAB in Fig. 10 demonstrate that the total input impedance angle is very close to zero when *R*_L_ is more than 5Ω. This means that the zero phase angle and ZVS condition are possible to achieve. The last curve in Fig. 10 shows that the output voltage increase linearly with *R*_L_. This means a fine constant output current is achieved. Response of the system when frequency changes in COC mode is similar to the COV mode.

**Figure 11:**
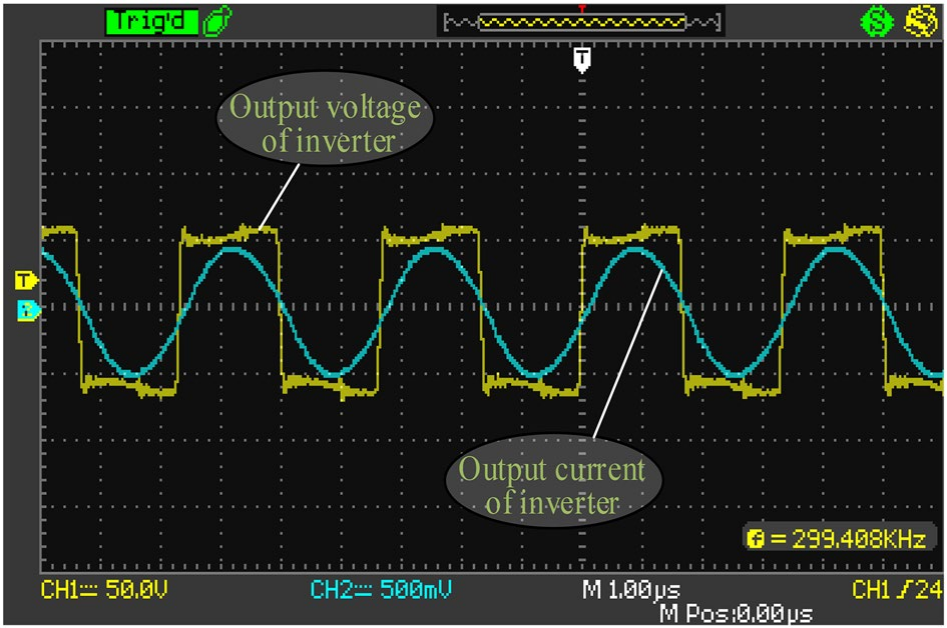
Output waveform of the inverter. (Obtained by oscilloscope, and the descriptive text is added by the “Microsoft Office Visio 2013” url: https://www.microsoft.com/zh-cn/microsoft-365/previous-versions/microsoft-visio-2013).

**Figure 12:**
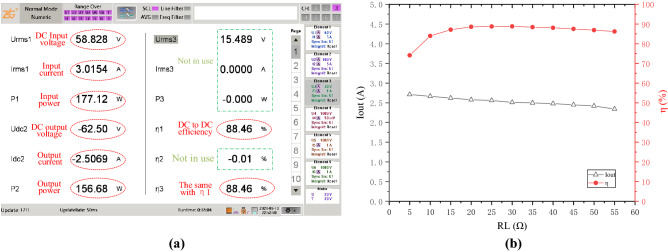
Experimental results in COC mode. (**a**) DC input and DC output power tested by ZLG Power Analyzer. (**b**) The tested output current and efficiency change with RL. ((**a**) is obtained by power analyzer, and the descriptive text is added by the “Microsoft Office Visio 2013” url: https://www.microsoft.com/zh-cn/microsoft-365/previous-versions/microsoft-visio-2013, (**b**) is created by origin 2018, url: https://www.0daydown.com/tag/originpro-2018).

The output waveform of the inverter taken by the oscilloscope is exhibited in Fig. 11. The current waveform slightly lags behind the voltage, indicating ZVS condition is achieved. The DC-to-DC efficiency of the COC system can always reach above 87%. Figure 12a shows an efficiency of 88.46% when *R*_L_ is 25Ω and *U*_in_ is 58.828 V. The black curve in Fig. 12b indicates the output current is relatively constant when *R*_L_ varies and *U*_in_ is set to about 58.828 V, and the red curve shows almost the same trend with the simulation results in the first diagram in Fig. 10. The difference between Figs. 10 and 12b is mainly because the energy loss of the inverter and the rectifier in Fig. 10 has not been taken into consideration. Experiments have also verified that the system efficiency can easily reach above 90% when the DC source provides a voltage more than 100 V.

**Figure 13:**
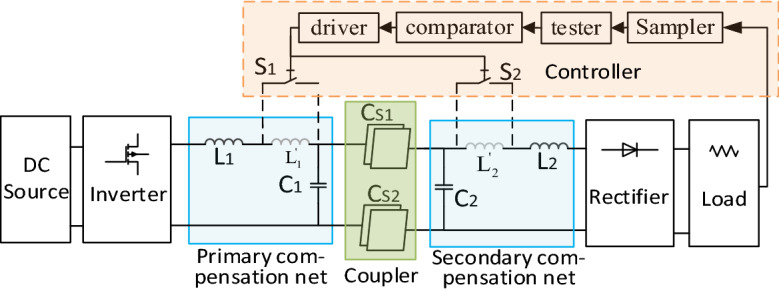
CPT with a control circuit for switching between constant output voltage and COC modes. (Created by the “Microsoft Office Visio 2013” url: https://www.microsoft.com/zh-cn/microsoft-365/previous-versions/microsoft-visio-2013).

**Figure 14:**
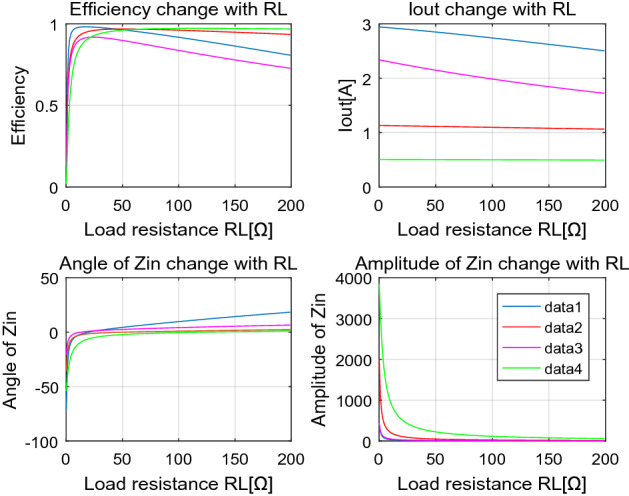
Comparison of output stability when compensated by different parameters or current gain Gi in COC mode. (Created by “matlab R2016a” url: https://ww2.mathworks.cn/products/matlab.html).

## Discussion

A. *Safety issues* The electromagnetic safety property of CPT system is often doubted. Some researches^9,29^ have been carried out to investigate it. Nevertheless, safety is a relevant definition. It is undeniable that the electric field in the area between couple plates and in the very nearby area is so high that it may exceed the criterion of the IEEE C95.1 standard^35^. However, the electric field decreases very rapidly with distance^19^. Safety can be ensured except for the dangerous areas. It is pointed out that the dangerous area is within 350 mm while the couple capacitance is only 2.8 pF and the voltage between plates reaches as high as 1.73 kV^19^. The couple capacitance can be enhanced through many approaches, like coating metallic plates with a very thin high permittivity material, and letting them rely on each other to shorten the distance between plates. Bigger couple capacitance will ensure a lower voltage stress on the plates, thus the dangerous area will be further restricted. Many other approaches can also be applied to ensure safety, like physical isolation to keep the organisms out of dangerous areas, newly designed configuration of the coupler to restrict the power emitted by couple plates.

B. *Switch between COV and COC mode* In some cases, switching between COV and COC is required. For example, the course of charging a battery is usually COC first, and COV at the end. According to the proposed decoupling method, the COV and COC system can be designed with a little difference in *L*_1_, *L*_2_ only. Thus, to switch between COV and COC modes, a control loop like Fig. 13 shows can be built. The inductor *L*_1_ and *L*_2_ can be divided into two parts in Fig. 13, *L′*_1_ and *L′*_2_ represent the inductance difference between COV and COC modes. The control loop contains a sampler, a tester, a comparator, a driver and two switches S_1_, S_2_. Suppose CPT system works in COC mode at first, and S_1_, S_2_ is open. When the voltage reaches a value near the full voltage of the battery, this signal will be sampled and then tested. This tested value will be compared with the predefined voltage point for switching charge mode and then a decision will be made to close S_1_, S_2_. It should be noted that a communication path needs to be established between the primary and secondary parts to transfer the control signal. Another way to switch between COV and COC mode is to change the operation frequency. Supposing that all compensation components are designed, so the frequency of COV and COC modes can be expressed as (24). When switching between COV and COC mode is required, it is just needed to switch the PWM signal between the frequency *f*_*COV*_ and *f*_*COC*_*.*
  24 $$\left\{ \begin{array}{*{20}l} f_{{{\text{COV}}}} = \frac{\omega }{2\pi } = \frac{1}{{2\pi \sqrt {L_{1} (C_{1} + C_{0} )} }} = \frac{1}{{2\pi \sqrt {L_{2} (C_{2} + C_{e} )} }} \\ f_{{{\text{COC}}}} = \frac{\omega }{2\pi } = \frac{{\sqrt {C_{2} + C_{{\text{M}}} } }}{{2\pi \sqrt {L_{1} (C_{1} C_{2} + C_{1} C_{{\text{M}}} + C_{2} C_{{\text{M}}} )} }} = \frac{{\sqrt {C_{1} + C_{{\text{M}}} } }}{{2\pi \sqrt {L_{2} (C_{1} C_{2} + C_{1} C_{{\text{M}}} + C_{2} C_{{\text{M}}} )} }}. \\ \end{array} \right.$$

C. *Output stability in response to different compensation parameters and current gain G*_*i*_ It is a common sense that parasitic resistance exists in each component in spite of sparing no effort to reduce it. The parasitic resistance would affect the input or output characteristics of the circuit more or less. The affection by different groups of system parameters and current gain *G*_i_ is evaluated through MATLAB simulation using the parameters listed in Table 3. The results are shown in Fig. 14. By comparing the curves in Fig. 14, it can be concluded that a smaller *G*_i_ will induce a more stable output current and efficiency.

## Conclusion

This paper introduces a newly designed load decoupling method that can achieve both COV and COC mode in double side LC compensated CPT. Through the analysis of basic circuit characteristics, the conditions for both two modes are determined. The proposed method has following three advantages:

1. The conditions indicate a very clear relationship between the compensation components;
2. The couple capacitors also participate in the resonant tanks;
3. The COC mode can theoretically reach zero phase angle condition, minimizing the imaginary power as much as possible, while the COV mode can’t.

Besides, an efficient model of double side LC compensated CPT is built, and the optimum load is calculated theoretically based on the model. Based on the constant output conditions and efficient model, the parameter design methodology is proposed. Results of both simulations and experiments demonstrate high agreement with the theoretical analysis. Finally, three practical issues are discussed, including electromagnetic safety, switching between the two modes, and stability of output with different groups of parameters. In future research work, we will concentrate on the reduction of parameter sensitivity and optimization of compensation net, efficiency improving scheme and stability control, and the mechanism of transferring power in seawater.
